# How much can we reduce delivery-related medical costs associated with maternal mortality? A nationwide cohort study from 2003 to 2021

**DOI:** 10.3389/fpubh.2025.1411534

**Published:** 2025-03-28

**Authors:** Jin Young Nam

**Affiliations:** Department of Healthcare Management, Eulji University, Seongnam, Republic of Korea

**Keywords:** maternal mortality, maternal death, medical costs, NHIS delivery cohort, pregnancy-related medical costs

## Abstract

**Objective:**

This study aims to examine the association between maternal mortality and childbirth-related medical costs using both unadjusted and adjusted models and to assess the potential reduction in delivery-related medical costs associated with maternal mortality in South Korea.

**Methods:**

This retrospective cohort study used data from the National Health Insurance Service Delivery Cohort Database of South Korea. A total of 7,171,578 participants were included. The outcome measured was delivery-related medical costs associated with maternal mortality. A Generalized Estimating Equation model with a log link and gamma distribution was used to estimate delivery-related medical costs.

**Results:**

The maternal death rates were 9.7 per 100,000 births. The adjusted mean delivery-related medical costs were approximately six times higher in cases with maternal death than in those without ($2,802 vs. $480, *p* < 0.0001). The total delivery-related medical costs for all women with maternal mortality were approximately $2 million, accounting for 0.06% of total delivery-related medical costs. Although this proportion is relatively small, 83% of the direct medical costs associated with maternal mortality among South Korean women were potentially reducible.

**Conclusion:**

This study found that maternal mortality is associated with significantly higher delivery-related medical costs, nearly six times those of non-maternal mortality cases. Therefore, policymakers should consider reducing costs and improving maternal health outcomes, expanding access to prenatal care for early risk detection and strengthen nationwide maternal health monitoring systems.

## Introduction

Maternal mortality remains a critical public health issue worldwide, despite substantial advancements in medical care. Most cases of maternal mortality are preventable and reducing maternal deaths has been a global health priority for decades (1, 2). However, maternal mortality rates have remained high in several high-income countries. For instance, in 2020, the maternal mortality ratio (MMR) in the United States and South Korea was 23.8 and 11.8 per 100,000 live births, respectively, ranking among the highest in high-income nations (3–6). Although maternal mortality is a relatively rare event in high-income countries, it imposes significant economic and social burdens on individuals and healthcare systems.

Among these burdens, the economic impact of maternal mortality is a crucial yet understudied aspect. The financial burden associated with maternal deaths affects not only the healthcare system but also the families and society at large. Despite this, studies on the economic costs of maternal mortality are scarce due to several limitations: (1) low maternal mortality rates in high-income countries leading to data scarcity (7), (2) a predominant focus on chronic diseases in medical cost research (8), (3) challenges in establishing a causal relationship between maternal mortality and medical costs, (4) a preventive approach in public health research (7), and (5) difficulties in cost comparisons across differences in healthcare systems (9).

While some studies have explored the indirect economic costs of maternal mortality, they were primarily conducted in low-and middle-income countries, such as those examining the household financial burden of maternal deaths in rural areas (10, 11). In high-income countries, a few studies have investigated the economic burden associated with severe maternal morbidity (SMM) (12, 13) or pregnancy-related complication such as preeclampsia, gestational diabetes, and ICU admissions (14–17). These studies have consistently demonstrated that women with SMM or pregnancy-related complications incur significantly higher medical costs compared to those without those. However, very few studies have directly examined the economic burden of maternal mortality itself, particularly in high-income setting.

Despite increasing recognition of this issue, limited research has quantified the direct medical costs of maternal mortality. One study estimated the economic impact of maternal mortality in the United States in 2019 at $30.8 million (8, 14), but no study has comprehensively analyzed the direct medical costs of maternal mortality using long-term, nationwide data in high-income countries.

Therefore, this study aims to examine the association between maternal mortality and childbirth-related medical costs using both unadjusted and adjusted models and to assess the potential reduction in delivery-related medical costs associated with maternal mortality in South Korea, based on a nationwide, 19-year population-based delivery cohort database.

## Methods

### Data source and study population

This population-based study used the database of the Korean National Health Insurance Service (NHIS), a single insurer of the entire country’s population, to which approximately 98% of all South Koreans are enrolled (18). The NHIS database retains data on the following: sociodemographic characteristics; healthcare utilization (received services and treatment costs); clinically determined diagnostic codes from the International Classification of Diseases, 10th revision (ICD-10); prescriptions with drug codes; days of prescription; daily dosages (18). The database uses de-identified join keys to link databases while ensuring patient anonymity (18). The study design was reviewed and approved by the Institutional Review Board of Eulji University (IRB Number: EU22-27). The requirement for informed consent was waived as the data did not contain identifiable information.

We extracted data from the NHIS claims databases for all women who delivered in medical institutions in South Korea between 1 January 2003, and 31 December 2021. Childbirth was identified using all inpatient records, including pregnancy-related diagnoses and vaginal or cesarean delivery procedures. The study population included women aged 15–49 years and those who gave birth between 1 January 2003, and 19 November 2021, so that the data on childbirth within 6 weeks postpartum could be analyzed. The study population comprised 7,203,924 deliveries. Notably, we excluded women who gave birth after November 19, 2021 (*n* = 7,415), had no healthcare institution delivery data (*n* = 7,836), or had no information due to missing data (*n* = 17,095). A total of 7,171,578 deliveries were included in this study.

### Delivery-related medical costs

Delivery-related medical costs were calculated from the claimed total direct medical costs during delivery hospitalization and the 6-week postpartum period. As the NHIS database does not include outpatient drug costs or uncovered healthcare service costs (such as uncovered treatments, medical administrations and injections, and nonstandard accommodations), the costs reported herein do not include those of the uncovered services.

To compare prices from different calendar years, costs were inflated to 2020 values using the South Korea Consumer Price Index for healthcare from the Bank of Korea by multiplying them by a year-specific inflation factor (19). To express costs in US dollars, they were converted from Korean won to US dollars using the annual exchange rates of the Ministry of Economy and Finance for each year (20).

### Maternal mortality

Maternal mortality was defined as the death of a woman during pregnancy or within 6 weeks postpartum (21). Maternal mortality included all-cause mortality because the cause of death was not available in the database.

### Covariates

The covariates included maternal sociodemographic and clinical factors. Sociodemographic factors included maternal age (<19, 19–24, 25–29, 30–34, 35–39, 40–44, or > 45 years), income level (quartile), type of insurance (self-employed insurance, employee insurance, or medical aid), and residential area (Seoul, metropolitan areas, small cities, or rural areas). Maternal clinical factors included the following: mode of delivery (spontaneous vaginal delivery, instrumental delivery, or cesarean section delivery); preterm birth (delivered at <37 vs. ≥37 weeks); parity (nulliparous or multiparous); multiple birth status (singleton vs. twin or more); adequacy of prenatal care (estimated by the Kessner Adequacy of Prenatal Care Index (22), which categorized adequate vs. inadequate, including intermediate, prenatal care); obstetric comorbidity [assessed by Bateman’s obstetric comorbidity index (23)]; type of hospital (general hospital with more than 500 beds, general hospital with 100–499 beds, hospital with 30–99 beds, and clinics with less than 30 beds); delivery year.

### Statistical analysis

We analyzed the distribution of maternal mortality and SMM according to maternal sociodemographic and clinical factors using descriptive statistics. We calculated the unadjusted mean delivery-related total medical costs and their 95% confidence intervals (CIs) to test their differences for childbirth with and without maternal mortality and SMM and all variables using the Kruskal–Wallis test. We used a generalized estimating equation (GEE) model with a log link, gamma distribution, and robust standard errors to estimate the mean delivery-related medical costs of maternal mortality and other variables, adjusted for covariates. We performed a stratified analysis using the GEE model to calculate the association between the adjusted delivery-related medical costs and maternal mortality by residential area. All statistical analysis was conducted using SAS 9.4 (SAS Institute, Inc., Cary, NC, United States). The level of significance was set at *p* < 0.05.

## Results

In total, 7,171,578 deliveries were included in this study. The maternal mortality rate was 9.7 per 100,000 deliveries. Women aged 35 years and older had a higher proportion of maternal death (35–39 years: 32.8%; 40–44 years: 6.1%; 45 years and older, 0.1%). Maternal mortality gradually decreased from 7.5% in 2003 to 3.2% in 2021 (Table 1).

**Table 1:** General characteristics of study population.

	Maternal mortality					
	Deceased		Survived		Total	
	*N*	(%)	No	(%)	No	(%)
Total	7,170,885	99.99	693	0.01	7,171,578	100
Maternal age						
<19	24,294	0.34	3	0.43	24,297	0.34
19–24	339,769	4.74	19	2.74	339,788	4.74
25–29	1,910,357	26.64	154	22.22	1,910,511	26.64
30–34	3,314,261	46.22	247	35.64	3,314,508	46.22
35–39	1,372,149	19.14	227	32.76	1,372,376	19.14
40–44	203,099	2.83	42	6.06	203,141	2.83
45+	6,956	0.10	1	0.14	6,957	0.10
Income level						
1Q	1,449,800	20.22	176	25.40	1,449,976	20.22
2Q	1,799,441	25.09	178	25.69	1,799,619	25.09
3Q	2,521,797	35.17	236	34.05	2,522,033	35.17
4Q	1,399,847	19.52	103	14.86	1,399,950	19.52
Type of insurance						
Self-employed insured	1,873,725	26.13	244	35.21	1,873,969	26.13
Employee insured	5,257,942	73.32	434	62.63	5,258,376	73.32
Medical aid	39,218	0.55	15	2.16	39,233	0.55
Residential area						
Seoul	1,453,088	20.26	142	20.49	1,453,230	20.26
Metropolitans	1,773,351	24.73	159	22.94	1,773,510	24.73
Small cities	3,479,580	48.52	345	49.78	3,479,925	48.52
Rural areas	464,866	6.48	47	6.78	464,913	6.48
Mode of delivery						
Spontaneous vaginal delivery	2,330,317	32.50	116	16.74	2,330,433	32.50
Instrumental delivery	2,016,484	28.12	125	18.04	2,016,609	28.12
Cesarean section delivery	2,824,084	39.38	452	65.22	2,824,536	39.39
Preterm birth						
No	6,998,961	97.6	655	94.52	6,999,616	97.6
Yes	171,924	2.40	38	5.48	171,962	2.40
Prenatal care						
Adequate	6,205,455	86.54	560	80.81	6,206,015	86.54
Inadequate	965,430	13.46	133	19.19	965,563	13.46
Parity						
Nulliparous	3,746,237	52.24	386	55.70	3,746,623	52.24
Multiparous	3,424,648	47.76	307	44.30	3,424,955	47.76
Multiple birth status						
Singleton	7,060,400	98.46	669	96.54	7,061,069	98.46
Twin or more	110,485	1.54	24	3.46	110,509	1.54
Obstetric comorbidities						
0	4,948,703	69.01	252	36.36	4,948,955	69.01
1+	2,222,182	30.99	441	63.64	2,222,623	30.99
Type of hospital						
General hospital (> 500 beds)	434,258	6.06	126	18.18	434,384	6.06
General hospital (100–499 beds)	760,444	10.60	120	17.32	760,564	10.61
Hospital (30–99 beds)	2,939,447	40.99	178	25.69	2,939,625	40.99
Clinics (<30 beds)	3,036,736	42.35	269	38.82	3,037,005	42.35
Year						
2003	385,661	5.38	52	7.50	385,713	5.38
2004	390,444	5.44	53	7.65	390,497	5.45
2005	387,726	5.41	41	5.92	387,767	5.41
2006	406,866	5.67	53	7.65	406,919	5.67
2007	455,942	6.36	54	7.79	455,996	6.36
2008	431,675	6.02	44	6.35	431,719	6.02
2009	413,729	5.77	50	7.22	413,779	5.77
2010	441,087	6.15	45	6.49	441,132	6.15
2011	443,138	6.18	46	6.64	443,184	6.18
2012	454,226	6.33	27	3.90	454,253	6.33
2013	407,474	5.68	40	5.77	407,514	5.68
2014	398,237	5.55	33	4.76	398,270	5.55
2015	399,697	5.57	31	4.47	399,728	5.57
2016	371,028	5.17	20	2.89	371,048	5.17
2017	326,434	4.55	16	2.31	326,450	4.55
2018	298,562	4.16	21	3.03	298,583	4.16
2019	276,217	3.85	21	3.03	276,238	3.85
2020	246,116	3.43	24	3.46	246,140	3.43
2021	236,626	3.30	22	3.17	236,648	3.30

The average total delivery-related medical costs with unadjusted all covariates for all participants were $1,157 (95% CI $1,156–$1,157); the costs in cases with maternal mortality were $7,634 (95% CI, $6,717–$8,551) and those in cases without maternal mortality were $1,156 (95% CI $1,156–$1,157) over 19 years (Table 2). The average medical costs varied significantly based on the covariates (*p* < 0.0001 for each) (Table 2).

**Table 2:** Unadjusted model for delivery costs on maternal mortality.

	Delivery medical costs for maternal mortality (2008.1~2021.11)				
	*N*	Mean cost (USD)	lower 95% CI	Upper 95% CI	*p*-value
Total	7,171,578	1,157	1,156	1,157	
Maternal death within 42 days before childbirth					<0.0001
Deceased	7,170,885	1,156	1,156	1,157	
Survived	693	7,634	6,717	8,551	
Maternal age					<0.0001
<19	24,297	998	991	1,005	
19–24	339,788	1,017	1,015	1,019	
25–29	1,910,511	1,028	1,027	1,028	
30–34	3,314,508	1,139	1,138	1,139	
35–39	1,372,376	1,358	1,357	1,360	
40–44	203,141	1,547	1,542	1,551	
45+	6,957	1,625	1,601	1,649	
Income level					<0.0001
1Q	1,449,976	1,158	1,157	1,159	
2Q	1,799,619	1,134	1,133	1,135	
3Q	2,522,033	1,155	1,154	1,156	
4Q	1,399,950	1,189	1,188	1,190	
Type of insurance					<0.0001
Self-employed insured	1,873,969	1,101	1,100	1,102	
Employee insured	5,258,376	1,177	1,177	1,178	
Medical aid	39,233	1,056	1,049	1,062	
Residential area					<0.0001
Seoul	1,453,230	1,158	1,157	1,159	
Metropolitans	1,773,510	1,180	1,179	1,181	
Small cities	3,479,925	1,153	1,152	1,153	
Rural areas	464,913	1,094	1,092	1,096	
Mode of delivery					<0.0001
Spontaneous vaginal delivery	2,330,433	848	847	848	
Instrumental delivery	2,016,609	1,031	1,030	1,032	
Cesarean section delivery	2,824,536	1,502	1,501	1,503	
Preterm birth					<0.0001
No	6,999,616	1,146	1,145	1,146	
Yes	171,962	1,605	1,599	1,612	
Prenatal care					<0.0001
Adequate	6,206,015	1,182	1,182	1,183	
Inadequate	965,563	993	992	995	
Parity					<0.0001
Nulliparous	3,746,623	1,221	1,220	1,222	
Multiparous	3,424,955	1,087	1,086	1,087	
Multiple birth status					<0.0001
Singleton	7,061,069	1,147	1,146	1,147	
Twin or more	110,509	1,811	1,804	1,818	
Obstetric comorbidities					<0.0001
0	4,948,955	1,065	1,065	1,066	
1+	2,222,623	1,360	1,359	1,362	
Type of hospital					
General hospital (>500 beds)	434,384	1,584	1,580	1,588	
General hospital (100–499 beds)	760,564	1,274	1,272	1,276	
Hospital (30–99 beds)	2,939,625	1,192	1,191	1,193	
Clinics (<30 beds)	3,037,005	1,033	1,032	1,033	
Year					<0.0001
2003	385,713	685	684	687	
2004	390,497	784	782	785	
2005	387,767	885	884	887	
2006	406,919	957	955	959	
2007	455,996	1,025	1,024	1,027	
2008	431,719	795	794	796	
2009	413,779	880	878	882	
2010	441,132	943	942	945	
2011	443,184	982	980	983	
2012	454,253	1,155	1,154	1,157	
2013	407,514	1,276	1,274	1,278	
2014	398,270	1,262	1,260	1,264	
2015	399,728	1,224	1,222	1,226	
2016	371,048	1,252	1,249	1,254	
2017	326,450	1,537	1,534	1,539	
2018	298,583	1,637	1,634	1,640	
2019	276,238	1,754	1,751	1,758	
2020	246,140	2,064	2,060	2,067	
2021	236,648	2,042	2,038	2,045	

The mean delivery-related total medical costs were adjusted for all covariates, and the association between the adjusted costs and maternal mortality was analyzed (Table 3). Patients with maternal mortality incurred significantly higher medical costs than those without. The adjusted mean (CI) delivery-related costs were $2,802 (95% CI $2,717–$2,889), which was 5.8-fold higher with maternal mortality than without maternal mortality. The total delivery-related medical cost for all women with maternal mortality {number with maternal mortality (*n* = 693) × adjusted mean cost of maternal mortality ($2,802)} was approximately $1.94 million, representing 0.06% of the total delivery-related medical costs. Moreover, maternal mortality-related costs have significantly increased in recent years, from $546 (95% CI $544–$548) in 2003 to $1,352 (95% CI $1,347–1,357) in 2021.

**Table 3:** Adjusted model for delivery medical costs on maternal mortality.

	Delivery medical costs				
	Parameter estimate	Mean cost (USD)	Lower 95% CI	Upper 95% CI	*p*-value
Intercept	6.1738	480	479	481	<0.0001
Maternal mortality					
Deceased	0.0000				
Survived	1.7643	2,802	2,717	2,889	<0.0001
Maternal age					
<19	−0.0143	473	470	476	<0.0001
19–24	−0.0104	475	473	477	<0.0001
25–29	0.0000				
30–34	0.0074	484	482	485	<0.0001
35–39	0.0066	483	482	485	<0.0001
40–44	0.0346	497	495	499	<0.0001
45+	0.047	503	498	509	<0.0001
Income level					
1Q	0.0001	480	479	481	0.8414
2Q	−0.002	479	478	480	<0.0001
3Q	−0.002	479	478	480	<0.0001
4Q	0.0000				
Type of insurance					
Self-employed insured	−0.0011	479	478	481	0.0008
Employee insured	0.0000				
Medical aid	−0.1075	431	429	434	<0.0001
Residential area					
Seoul	0.0000				
Metropolitans	0.0273	493	492	495	<0.0001
Small cities	0.0084	484	483	485	<0.0001
Rural areas	0.0108	485	484	487	<0.0001
Mode of delivery					
Spontaneous vaginal delivery	0.0000				
Instrumental delivery	0.1068	534	533	535	<0.0001
Cesarean section delivery	0.4712	769	767	771	<0.0001
Preterm birth					
No	0.0000				
Yes	0.0479	504	502	505	<0.0001
Prenatal care					
Adequate	0.0000				
Inadequate	−0.0125	474	473	475	<0.0001
Parity					
Nulliparous	0.1069	534	533	535	<0.0001
Multiparous					
Multiple birth status					
Singleton	0.0000				
Twin or more	0.0486	504	502	506	<0.0001
Obstetric comorbidities					
0	0.0000				
1+	0.0548	507	506	509	<0.0001
Type of hospital					
General hospital (>500 beds)	0.2341	607	605	609	<0.0001
General hospital (100–499 beds)	0.148	557	555	558	<0.0001
Hospital (30–99 beds)	0.0000				
Clinics (<30 beds)	−0.0595	452	451	453	<0.0001
Year					
2003	0.0000				
2004	0.1294	546	544	548	<0.0001
2005	0.2836	637	635	640	<0.0001
2006	0.3606	688	686	691	<0.0001
2007	0.435	742	739	744	<0.0001
2008	0.187	579	577	581	<0.0001
2009	0.2879	640	638	642	<0.0001
2010	0.3603	688	686	691	<0.0001
2011	0.4085	722	720	725	<0.0001
2012	0.561	841	838	844	<0.0001
2013	0.648	918	914	921	<0.0001
2014	0.6351	906	903	909	<0.0001
2015	0.5968	872	869	875	<0.0001
2016	0.6079	882	878	885	<0.0001
2017	0.8006	1,069	1,065	1,073	<0.0001
2018	0.8533	1,127	1,123	1,131	<0.0001
2019	0.9104	1,193	1,188	1,198	<0.0001
2020	1.063	1,390	1,384	1,395	<0.0001
2021	1.0355	1,352	1,347	1,357	<0.0001

Adjusted for maternal age, income level, type of insurance, residential area, mode of delivery, preterm birth, prenatal care, parity, multiple births, obstetric comorbidities, type of hospital, and year.

## Discussion

This study demonstrated a strong association between maternal mortality and delivery-related medical costs, with a nearly six-fold increase in costs among women who experienced maternal mortality. The results provide a detailed breakdown of these costs, highlighting that maternal mortality cases incurred significantly higher expenses across all periods. These findings align with previous studies on severe maternal morbidity (SMM), which have shown increased medical costs associated with adverse maternal outcomes (12). Consequently, over $1.6 million was spent from 2003 to 2021 on excess delivery-related medical costs due to maternal death, as calculated using adjusted cost estimates (693 cases × $2802 – 693 cases × $480). Importantly, approximately 83% ($1.6 million of $1.94 million) of the maternal mortality delivery-related medical costs could have been avoided through the prevention of mortality-related complications.

While the association between maternal mortality and medical costs is clear, the cause of the high cost is insufficiently understood, perhaps because maternal mortality is demographically rare and difficult to study. Consistent with this, a World Health Organization (WHO) study on maternal mortality did not estimate direct medical costs and included only low-income countries (24).

At 0.06% of the overall delivery-related medical costs, the proportion of the total delivery medical costs attributable to maternal mortality may seem small. This relates to the absolute number of maternal deaths, which is relatively low in South Korea because of its total fertility rate, which, at 0.81 children per woman, is the lowest in the world (25). Therefore, although the MMR in South Korea in 2022 is higher than that of the Organization for Economic Co-operation and Development (OECD) members (12 vs. 9 deaths per 100,000 live births in South Korea vs. OECD countries) (26), the absolute number of maternal deaths is extremely small. Furthermore, the burden of maternal mortality extends beyond direct medical costs to include the potential years of lost life, the statistical value of those years, and their impact on surviving family members. Thus, further studies are required to estimate the total burden of maternal mortality. As 83% of the direct medical costs associated with maternal mortality in South Korean women were potentially reducible, maternal health promotion can potentially improve maternal health outcomes and prevent tragic events.

Interestingly, the medical costs associated with maternal mortality have increased significantly in recent years. Although the reasons for this remain unknown, several mechanisms have been proposed. First, delivery-related medical costs may have been changed by the new fee-for-service policies. The Korean government supported several fertility-related policies to encourage the expansion of healthcare coverage. Since January 2005, spontaneous vaginal delivery has been free of charge. In the 4 years from its universal adoption for prospective payment in July 2012 to June 2016, cesarean section delivery accounted for 20% of the total out-of-pocket costs paid. Since July 2016, it has accounted for 5% of the total delivery-related costs. When the South Korean government implemented a financial support policy, out-of-pocket medical costs for cesarean section delivery dropped from 100 to 5%, reducing the financial burden on maternities. Therefore, pregnancies and obstetricians might choose their delivery by cesarean section more easily if needed. Nevertheless, total costs were not reduced but increased with respect to the consumer price inflation rate.

Moreover, the rate of cesarean section deliveries in South Korea has gradually increased, which is related to the medical costs of childbirth. According to OECD statistics, the rate of cesarean section deliveries from 2003 to 2020 dramatically increased from 36.5 to 53.8% in South Korea, the second-highest rate of cesarean section delivery in 2020 among OECD countries (27). A previous study showed that cesarean delivery is a high-risk factor for maternal mortality, and a report from a WHO Health Organization global survey involving nine Asian countries showed higher rates of cesarean section to be associated with a higher MMR (28). A similar finding was reported in a high-income country (29). Therefore, these numbers indicate that delivery-related medical costs may increase due to increasing C-section deliveries.

Finally, the South Korean government implemented several childbirth encouragement policies, including financial and service support measures such as iron supplementation (2008), vouchers for prenatal care and childbirth (2011), reduced out-of-pocket (OOP) medical costs for high-risk pregnancies (2015), and insured coverage of assisted reproductive technology. Direct or indirect improvement in maternal health and reduced financial burdens may have led to reduced OOP costs associated with maternal mortality in recent years. However, increasing new policies related to encouraging pregnancy may raise total medical costs because the utilization of healthcare services and accessibility to obstetricians may be better.

### Strengths and limitations

This study has several strengths. First, to the best of our knowledge, no other investigation has estimated the association between maternal mortality and delivery-related direct medical costs using a nationally representative database that includes all women who delivered in South Korea during an extended 19-year period. While several studies have examined the relationship between delivery costs and one of these conditions, none have estimated the costs regarding maternal mortality. Second, the endpoints were adjusted for numerous covarying demographic and obstetric factors, allowing for the detection of significant differences in the diverse case mix. Third, this study provides considerable data to support future studies on the association between maternal health outcomes and medical costs and the disease burden of maternal mortality and morbidity in various segments of the delivery population.

This study has some limitations. First, as the NHIS delivery cohort database does not include information on healthcare services not covered by insurance and policies affecting covered services that changed during the 19-year span of the study, some costs were inconsistently captured. For instance, the coverage of specialist medical service fees changed in January 2018, and the coverage of some non-standard hospital accommodations changed in July 2019. Consequently, total medical costs may have been underestimated. Second, maternal death included all-cause mortality within 42 days of childbirth. Thus, as the NHIS Delivery Cohort database did not include information on cause-specific mortality, it may have included incident- or accident-caused mortality. Further studies are necessary to link the government’s cause-specific mortality database with the NHIS Delivery Cohort database. Third, as the NHIS Delivery Cohort database uses revised ICD-10 codes that do not include procedure codes, we converted the ICD-10 codes for procedures, which may have made the identification of procedural cases less accurate.

## Conclusion

This study found that maternal mortality is associated with significantly higher delivery-related medical costs, nearly six times those of non-maternal mortality cases. Approximately 83% of these costs ($1.6 million) were potentially reducible, emphasizing the need for improved maternal health interventions. This study provides the first nationwide, long-term analysis of the direct medical costs of maternal mortality in a high-income country, highlighting its economic burden on both individuals and the healthcare system. To reduce costs and improve maternal health outcomes, policymakers should expand access to prenatal care for early risk detection and strengthen nationwide maternal health monitoring systems. Additionally, further research is needed to explore the broader economic impact of maternal mortality. By implementing these measures, maternal deaths can be reduced, and healthcare expenditures can be optimized, benefiting both individuals and society.

## Data Availability

Publicly available datasets were analyzed in this study. This data can be found at: https://nhiss.nhis.or.kr/.
